# Supplementary material for: Use of Almond Skins to Improve Nutritional and Functional Properties of Biscuits: An Example of Upcycling
Source: Foods. 2020 Nov 20;9(11):1705. doi: 10.3390/foods9111705 (PMC7699943; doi:10.3390/foods9111705)
Supplement: Supplementary file 1 [file foods-09-01705-s001.pdf]

foods

Supplementary Table S1. Regression equation, linear range, correlation coefficient (R^2), limit of detection (LOD), and limit of quantification (LOQ) of the HPLC analysis of the phenolic compounds quantified in the experimental biscuits.

	Regression equation	Linear range (mg/mL)	R^2	LOD (µg/mL)	LOQ (µg/mL)
Vanillic acid	y = 83450x - 163.97	0.005 – 0.5	0.9997	0.277	0.832
Syringic acid	y = 58126x + 7.647	0.005 – 0.5	1	0.413	1.239
p-Coumaric acid	y = 83450x + 163.97	0.005 – 0.5	0.9999	0.485	1.456
Ferulic acid	y = 65426x + 16.861	0.005 – 0.5	1	0.286	0.858
Sinapic acid	y = 77219x -95.354	0.005 – 0.5	0.9994	0.307	0.920
p-Hydroxybenzoic acid	y = 104370x + 24.846	0.005 – 0.5	0.9964	0.225	0.676
Protocatechuic acid	y = 59729x + 64.792	0.005 – 0.5	0.9997	0.406	1.217
(+)-Catechin	y = 8357x + 28.623	0.005 – 0.5	0.9998	0.384	1.152
(-)-Epicatechin	y = 12384x - 24.444	0.005 – 0.5	0.9999	0.259	0.778
